# Comparison of comet-based approaches to assess base excision repair

**DOI:** 10.1007/s00204-023-03543-y

**Published:** 2023-06-22

**Authors:** Congying Zheng, Sergey Shaposhnikov, Andrew Collins, Gunnar Brunborg, Florin Oancea, Frederik-Jan Van Schooten, Roger Godschalk

**Affiliations:** 1 Department of Pharmacology and Toxicology, NUTRIM School of Nutrition and Translational Research in Metabolism, Maastricht University, 6200 Maastricht, Netherlands; 2 Norgenotech AS, 64/66, 0379 Ullernchassern, Oslo Norway; 3 Oslo Cancer Cluster, 64/66, 0379 Ullernchausseen, Oslo Norway; 4 National Institute for Research & Development in Chemistry and Petrochemistry, Splaiul Independenței 202, București, Romania

**Keywords:** DNA repair, Comet assay, Genotoxicity, In vitro repair assay, Cellular repair assay

## Abstract

DNA repair plays an essential role in maintaining genomic stability, and can be assessed by various comet assay-based approaches, including the cellular repair assay and the in vitro repair assay. In the cellular repair assay, cells are challenged with a DNA-damaging compound and DNA damage removal over time is assessed. In the in vitro repair assay, an early step in the repair process is assessed as the ability of a cellular extract to recognize and incise damaged DNA in substrate nucleoids from cells treated with a DNA-damaging compound. Our direct comparison of both assays in eight cell lines and human peripheral blood lymphocytes indicated no significant relationship between these DNA repair assays (*R*^2^ = 0.084, *P* = 0.52). The DNA incision activity of test cells measured with the in vitro repair assay correlated with the background level of DNA damage in the untreated test cells (*R*^2^ = 0.621, *P* = 0.012). When extracts were prepared from cells exposed to DNA-damaging agents (10 mM KBrO_3_ or 1 µM Ro 19–8022 plus light), the incision activity was significantly increased, which is in line with the notion that base excision repair is inducible. The data presented suggest that the two assays do not measure the same endpoint of DNA repair and should be considered as complementary.

## Introduction

The comet assay (also known as single-cell gel electrophoresis) is a simple assay used for detecting DNA damage and DNA repair (Møller et al. 2020; Vodenkova et al. 2020). The assay is most often used to detect DNA damage but was initially developed to study the repair of ionizing radiation-induced strand breaks over time (Ostling and Johanson 1984; Singh et al. 1988). Among later developments of the assay, the cellular repair assay, or challenge assay, has been useful in monitoring the removal of damage with time, giving information about the overall process of repair, *i.e*., up to and including ligation. Apart from strand break rejoining, this approach can be applied to study the removal of altered bases via base excision repair (BER), by including digestion with a lesion-specific enzyme such as endonuclease III (Endo III) or formamidopyrimidine DNA glycosylase (Fpg) (Collins 2017; Gielazyn et al. 2003). BER is a complex multistep process, which includes (i) removal of the damaged base by a specific glycosylase that cleaves the bond between the base and the deoxyribose creating an apurinic or apyrimidinic site (AP site); (ii) cleavage of the DNA at the AP site by an AP endonuclease; (iii) ‘cleaning’ of the gap by the 5’ to 3’ exonuclease activity of DNA polymerase, followed by insertion of the correct nucleotide(s) using the complementary strand as a template; and (iv) sealing of the gap by DNA ligase (Krokan and Bjørås 2013). The cellular repair assay reflects the complete repair process; however, it is not ideal as a biomarker assay as it requires incubation of freshly isolated cells (Duthie et al. 2002), and human biomonitoring samples are often frozen. An alternative assay was developed in which extracts are made from unexposed test cells. Nucleoids prepared from substrate cells are then treated with the cell extract, and its DNA repair activity is estimated from incisions producing DNA breaks detected in the comet assay. Repair of DNA damage caused by different agents can be quantified with this method (Collins et al. 1994, 1995, 2001; Collins 2014; Langie et al. 2011; Vodenkova et al. 2020). The first application of the i*n vitro* repair assay measured BER activity of human cell extracts (Collins et al. 1994). Later, nucleotide excision repair (NER) was measured by the same approach (Langie et al. 2006). The assay was applied in clinical and biomonitoring settings. For instance, 70 pairs of colorectal carcinoma and adjacent healthy tissues were assessed for BER and NER activity; the assay proved to be suitable for high-throughput screening (Slyskova et al. 2012). In another example, Stoyanova and co-workers tested the recognition and incision of 8-oxoguanine by cell extracts from lymphocytes to obtain information on DNA repair in chronic renal failure patients undergoing hemodialysis (Stoyanova et al. 2014). In a nutritional intervention study with healthy subjects, BER activity was increased by consumption of kiwifruit as a supplement to their daily diet (without any increase in levels of OGG1 or APE1 mRNA) (Collins et al. 2003). A larger nutritional study proposed that the positive heath effect of fruit intake could increase the base and nucleotide excision repair ability (Slyskova et al. 2014).

Although the cellular repair assay and the in vitro repair assay have both been used to assess DNA repair in clinical and molecular epidemiological settings, a direct comparison of both approaches has never been performed and it is not clear whether they are directly comparable. We, therefore, made a study of both approaches in eight cell lines and in peripheral blood mononuclear cells (PBMCs).

## Materials and methods

### Chemicals

Chemicals and reagents used for the in vitro repair assay were purchased from Sigma Aldrich (Heidelberg, Germany); culture medium and reagents for cell culture were purchased from Biowest AS. Lymphoprep was purchased from Fresenius Kabi Norge As. Trypan blue solution 0.4% was purchased from Gibco Company (Thermo Fisher Scientific).

### Cells

PBMCs were isolated from venous blood collected in vacutainer tubes with EDTA as anticoagulant from five non-smoking, healthy volunteers (25–35 years, two men and three women) under ethical committee approval. The blood sample was diluted in a 15 ml plastic tube at a volume ratio of 1:1 with sterile PBS, underlayed with the same volume of Lymphoprep, and centrifuged at 250 × g for 30 min at 4 °C with the brake off. PBMCs were isolated from the interface between PBS and Lymphoprep, washed with PBS, centrifuged (250 × g, 5 min at 4 °C), and resuspended in 1 ml sterile PBS.

The following eight cell lines were also used in these experiments: MCF-7 (human breast cancer cell line), LNCaP (human prostate adenocarcinoma cell line), TK-6 (human lymphoblastoid cell line), HepG2 (human hepatocellular carcinoma cell line), V79-4 (Chinese hamster lung fibroblast cell line), Caco2 (human colorectal adenocarcinoma cell line), HeLa cells (human cervical cell line), and LLC-PK1 (porcine kidney cell line); We selected these cell lines, because we used them in a previous publication (Zheng et al. 2023). Moreover, the various cell lines will have a range of BER activity which improves the comparison of the approaches to assess repair. Lastly, these cell lines are widely used in biomedical research. All cells were grown in appropriate medium according to the protocol provided by the ATCC; they were incubated at 37 °C in a humidified incubator with a 5% CO_2_ atmosphere.

### Designation of cells

**Fig. 1:**
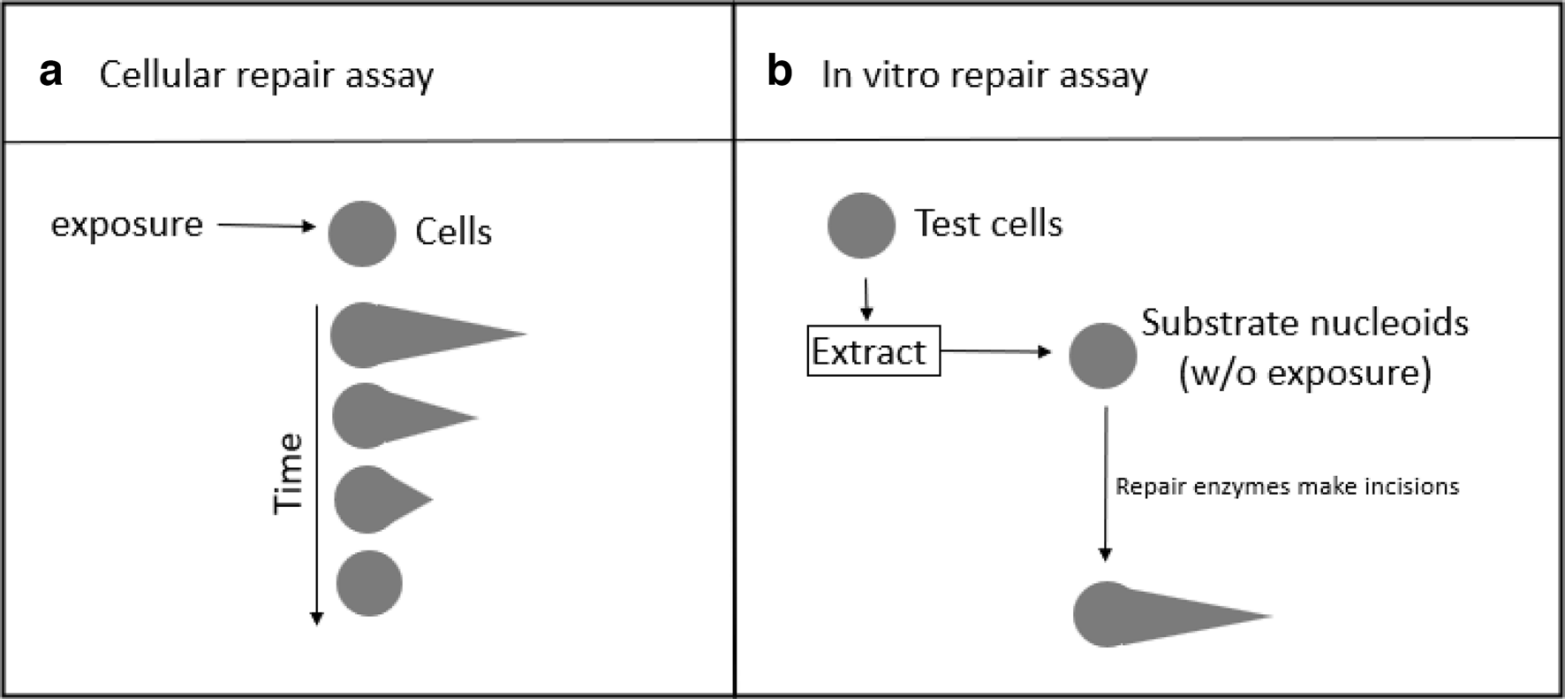
Principle of the two comet assay-based approaches to measure DNA repair. **A** *Cellular repair assay*: cells are exposed to DNA-damaging compounds (*e.g.*, KBrO_3_ or Ro19-8022 plus light). During subsequent repair incubation, DNA damage is measured with the Fpg comet assay at various time points after exposure to monitor DNA damage removal. **B** In vitro* repair assay*: extracts are made from test cells. The DNA repair activity of the extracts is measured by incubation with gel-embedded substrate nucleoids containing large amounts of DNA damage, resulting from prior exposure to KBrO_3_ or Ro19-8022 plus light. DNA repair enzymes in the test cell extracts recognize and incise the damaged sites, leading to DNA strand breaks. Subsequent electrophoresis leads to migration of DNA and higher % tDNA is indicative of more repair

For clarity, throughout this paper, the cells providing the substrate nucleoids are referred to as ‘substrate cells’, and the cells from which extracts are prepared are referred to as ‘test cells’ (Fig. 1).

### *Exposure of cells to Ro 19–8022 plus light or to KBrO*_*3*_

PBMCs and TK-6 cells were suspended in RMPI-1640 with 10% fetal bovine serum and placed in Petri dishes at a concentration of 2.5 × 10^5^ cells/ml. 1 ml of cell suspension was used as control, and the remaining cells in the Petri dish were placed on ice and subsequently treated by adding photosensitizer Ro 19–8022 (a gift from F. Hoffmann-La Roche) at 1 µM and exposing to visible light (33 cm from a 500 W tungsten halogen source) for 5 min. Alternatively, cells were incubated with 10 mM KBrO_3_ in appropriate cell medium for 1 h at 37 °C. After treatment, cells were washed with PBS, centrifuged (250 × g, 5 min at 4 °C), and resuspended in 1 ml sterile PBS.

Adherent cells were seeded into 24–well plates and allowed to grow to 70–85% confluence. Samples were treated with KBrO_3_ or incubated with Ro 19–8022 plus light as above. After treatment, cells were detached with trypsin–EDTA, washed with PBS, centrifuged (250 × g, 5 min at 4 °C) and resuspended in 1 ml sterile PBS.

### Cytotoxicity test

Trypan blue tests (Strober 2015) were conducted in parallel with the comet assay to assess cytotoxicity before and after exposure to KBrO_3_ or to Ro 19–8022 plus light. In all cases, viability was higher than 85%.

### Comet assay for strand breaks

The protocol of Collins (Collins et al. 2023) was applied with minor modifications. Cell suspensions were mixed with 0.7% low-melting-point agarose (LMPA) for the preparation of gels. After solidification, the embedded cells were lysed at 4 °C overnight (lysis solution: 2.5 M NaCl, 100 mM EDTA-Na_2_, 10 mM Tris base, pH 10 and 1% Triton X-100 added just before use). Slides were then placed in alkaline electrophoresis solution (0.3 M NaOH, 1 mM EDTA-Na_2_, pH > 12) for 20 min at 4 °C for unwinding and then electrophoresed in the same solution for 20 min at a voltage gradient of 0.8 V/cm across the platform in a horizontal electrophoresis chamber (Bio-Rad, Richmond, CA, USA). Finally, the slides were rinsed once with cold PBS (1 × , pH = 7.4), twice washed in cold distilled water and left to dry. After drying, slides were stained with 1 µM SYBR™ Gold for 30 min in the dark and then rinsed twice in distilled water.

The semi-automated image analysis system (Comet Assay IV; Perceptive Instruments) was used to evaluate 50 comets per gel. The percentage of DNA in the tail (% tDNA) was the descriptor used, and the median value of % tDNA from 100 comets was used to measure DNA damage for each condition.

### Fpg-modified comet assay

Fpg was produced by Norgenotech AS, Norway, and was the same enzyme (made in one batch) as used by the European Comet Assay Validation Group (ECVAG) (Godschalk et al. 2013). Aliquots were diluted tenfold with Fpg reaction buffer (40 mM HEPES, 0.1 M KCl, 0.5 mM EDTA-Na_2_, 0.2 mg/mL BSA, pH = 8) with the addition of 10% glycerol), and stored at – 80 °C. For each experiment, an aliquot was diluted with 30 ml of Fpg reaction buffer, reaching a final dilution of 60,000 times from the original crude preparation (0.5 µg/ml total protein). For enzyme treatments, the slides were washed with the enzyme reaction buffer three times after lysis, for 5 min each at 4 °C. The slides were then placed on a plastic rack, 50 µl Fpg solution or the Fpg reaction buffer (controls) was added to each gel, and a 22 × 22 mm coverslip was placed on top. Then the rack was transferred to a pre-heated moist box and placed in an incubator for 1 h at 37 °C. After incubation, the slides were placed at 4 °C in a cold room to terminate the Fpg reaction. The coverslips were removed, and all slides were transferred to the electrophoresis tank; subsequent steps were as for the standard comet assay for strand breaks. Net Fpg-sensitive sites were estimated by subtracting % tDNA for buffer incubation from % tDNA for Fpg incubation.

### In vitro* repair assay*

#### *Preparation of test cell extracts*

The protocol of Vodenkova et al. (2020) was applied. Samples of all cell types, without any genotoxic treatment, were centrifuged at 250 × g for 5 min at 4 °C. The supernatant was removed, and the pellet was washed with PBS, resuspended once more in cold PBS, and centrifuged at 2,000 × g for 5 min at 4 °C. The supernatants were removed as much as possible and discarded. 50 µl of extraction buffer A (45 mM HEPES, 0.4 M KCl, 1 mM EDTA-Na_2_, 0.1 mM DTT, 10% (vol/vol) glycerol, adjusted to pH = 7.8 using 6 M KOH) was added to each pellet of 5 × 10^6^ cells on ice. Samples were vortexed vigorously, snap-frozen in liquid nitrogen and then immediately thawed again. To each 50 µL aliquot, 15 µL of buffer A/1% Triton® X—100 was added and vortexed for 5 s and left for 10 min on ice. Finally, samples were centrifuged at 15,000 × g for 5 min at 4 °C to remove cell debris. The supernatant was collected in a new microtube and stored at –80 °C. For the experiment to test BER inducibility, three cell types (MCF-7, HepG2, and TK-6) were incubated with 10 mM KBrO_3_ for 1 h or treated with 1 µM Ro 19–8022 plus light for 5 min, before making test cell extracts following the same protocol.

#### Preparation of nucleoid substrates

Near-confluent cultures of TK-6 cells were incubated for 1 h with 10 mM KBrO_3_ or treated with 1 μM Ro 19–8022 plus light, to induce 8-oxoguanine as the substrate for BER. Half of the cells from each culture were suspended in freezing medium and stored at –80 °C, and the remainder were used immediately as fresh substrates; fresh substrates were used for the subsequent experiments.

#### Repair incubation

Nucleoid substrates (non-exposed and exposed) embedded in agarose, were incubated with reaction buffer only (controls) or with test cell extracts in reaction buffer as described above (50 µl on each gel); 22 × 22 mm coverslips were placed on top and the rack was transferred to a pre-warmed moist box and placed in an incubator for 1 h in 37 °C. Subsequent steps were the same as for the Fpg-modified comet assay for DNA damage (oxidized bases).

### Data analysis

Statistical analysis was conducted using IBM SPSS Statistics 21.0 and Excel 2021. The normality distribution of the experimental results was tested by the Kolmogorov–Smirnov test, followed by ANOVA with Dunnett's post hoc test for differences between groups. If the data did not fit normal distribution, the Whitney U test was used. All statistical tests were conducted at a 95% confidence level.

## Results

### In vitro* repair activity of test cell extracts from different cell lines*

**Fig. 2:**
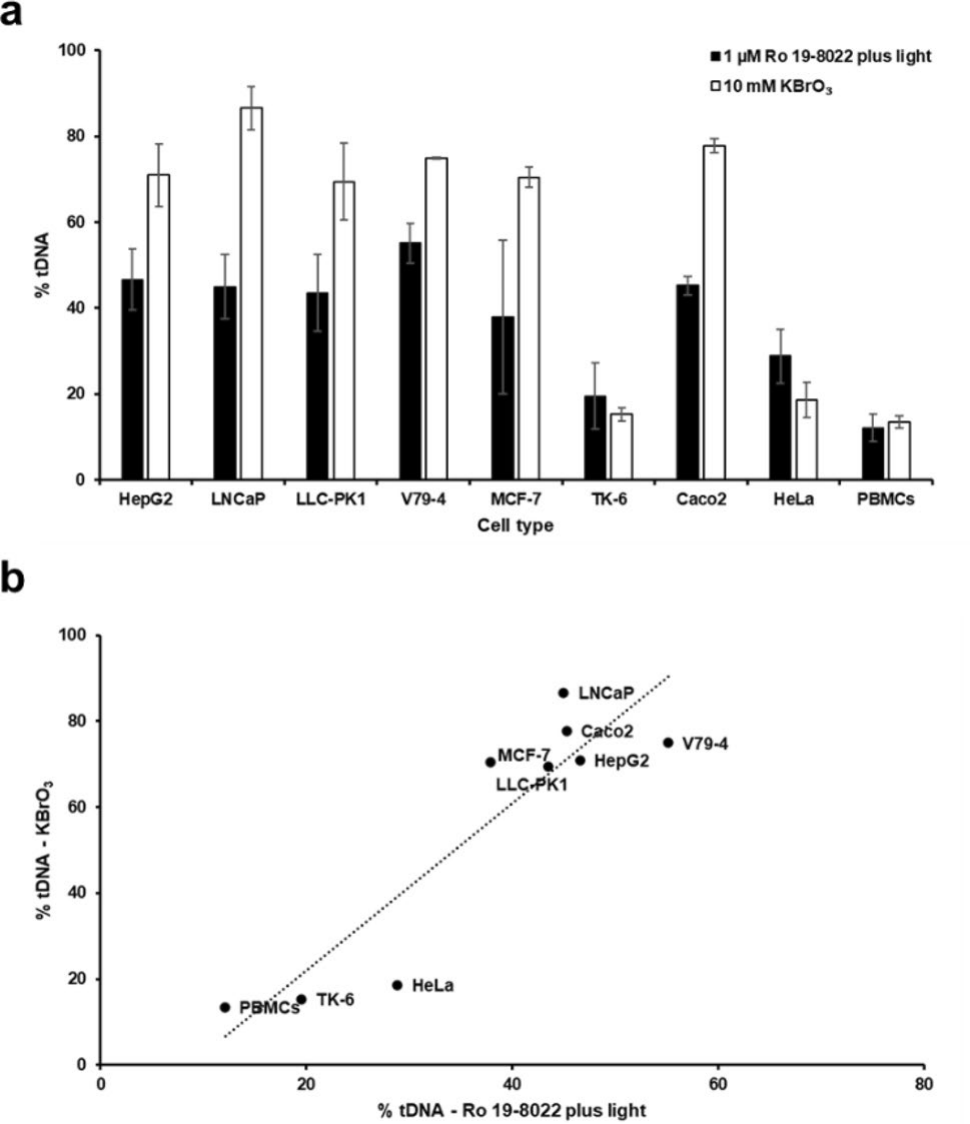
Incision activities of different test cell extracts on fresh substrate nucleoids from cells exposed to KBrO_3_ or to Ro 19–8022 plus light. **a** Mean values of three independent experiments (± SD indicated by error bars); **b** correlation between the incision activities shown in **a** (*R*^2^ = 0.829, *P* < 0.001)

**Fig. 3:**
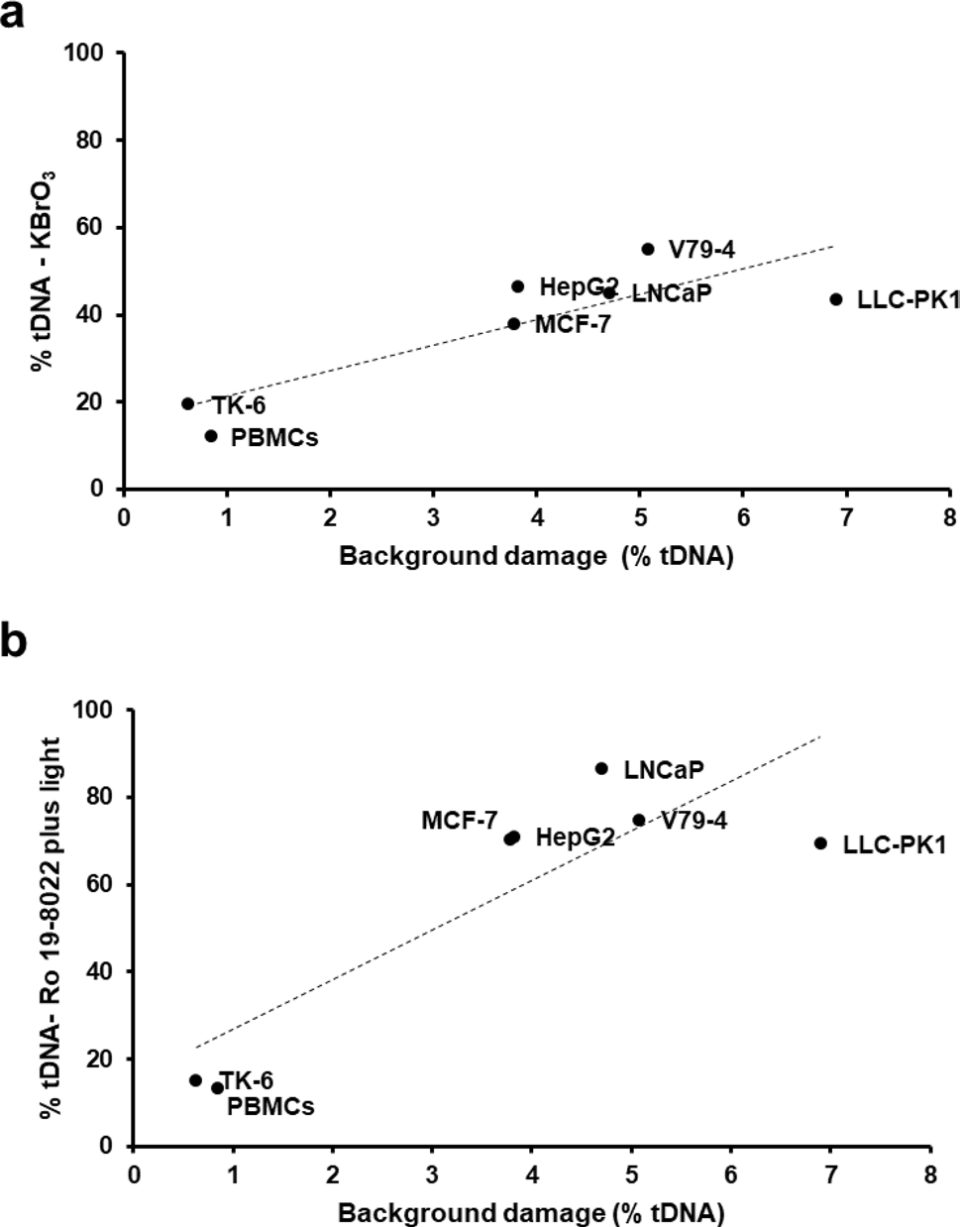
Correlation between incision activities in extracts from the different test cells, and the background damage in the corresponding (unexposed) cells. **a** Substrate cells exposed to KBrO_3_ (10 mM, 1 h) (*R*^2^ = 0.723, *P* < 0.05). **b** Substrate cells exposed to Ro 19–8022 (1 µM) plus light (*R*^2^ = 0.741, *P* < 0.05)

The various cell lines were chosen under the assumption that they would vary in their in vitro DNA repair activity (The same cell lines (V79-4, LNCaP, LLC-PK1, MCF-7, HepG2, TK-6, Caco2, HeLa) used in the preliminary experiment, which vary in their in vitro DNA repair activity, the data have been included into Fig. 2). Indeed, DNA incision activities of the test cell extracts (expressed as increase in net % tDNA after incubation for 60 min) varied from < 20% in TK-6 cells and PBMCs, to > 80% in LNCaP cells (Fig. 2a). The same dilutions of the various test cell extracts were used, and substrate cells were always TK-6. Similar relative incision activities of the test cell extracts were found whether the substrate cells were treated with 10 mM KBrO_3_, or with 1 µM Ro 19–8022 plus light (Fig. 2a). There was a significant correlation between incision activities of the nine test cell types with the two damaging agents (*R*^2^ = 0.829, *P* < 0.001) (Fig. 2b). We observed a strong correlation between DNA repair activity of the extracts on treated TK-6 cells, and the DNA damage in the corresponding (unexposed) test cells (*R*^2^ = 0.723, *P* < 0.05; *R*^2^ = 0.741, *P* < 0.05, for KBrO_3_ and Ro 19–8022 plus light, respectively) (Fig. 3). This suggests that the background damage in itself has a positive effect on higher DNA repair activity.

### *Cellular repair in different cell lines after exposure to KBrO*_*3*_*, or to Ro 19–8022 plus light*

We previously studied (Zheng et al. 2023) cellular repair in the same cell lines (V79-4, LNCaP, LLC-PK1, MCF-7, HepG2, TK-6, Caco2, HeLa) and PBMCs, treating them with either KBrO_3_ or with Ro 19–8022 plus light and estimating repair rate as the half-time for removal of damage. Data from these experiments were used in this paper to make the comparison of repair rates between the cellular and in vitro repair assays.

### *Direct comparison between cellular repair and *in vitro* repair assays*

**Fig. 4:**
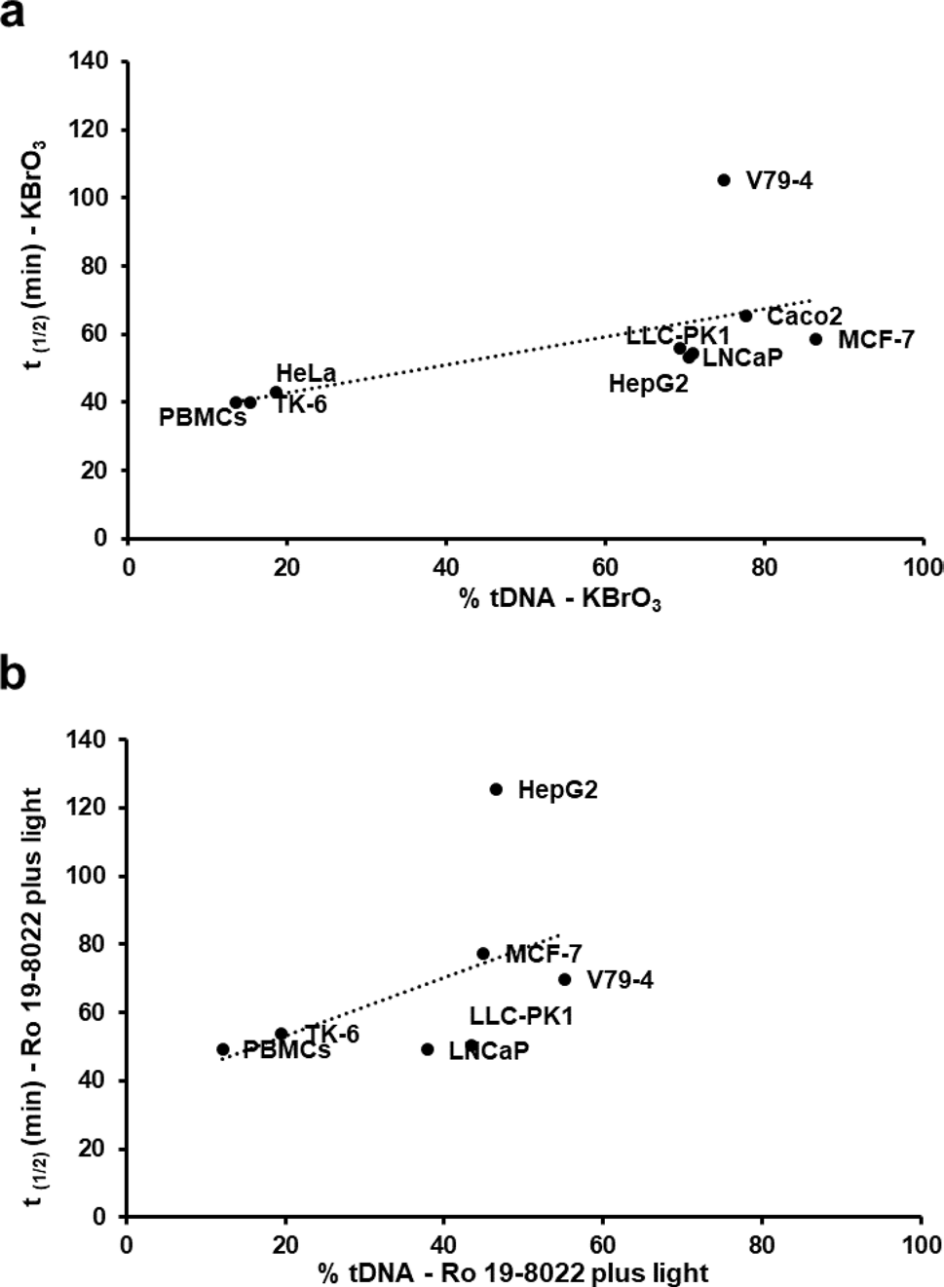
Correlations between rates of repair estimated by means of the two different assays. Half-life (t _1/2_) estimated with the cellular repair assay (y-axis), and incision activity (increase in % tDNA) estimated with the in vitro repair assay (x-axis). Cells for cellular assay or substrate cells for in vitro assay were treated with (**a**) KBrO_3_ for 1 h; or (**b**) Ro 19–8022 plus light for 5 min. Data for cellular repair are taken from our last publication (Zheng et al. 2023), but these two graphs in Fig. 4 are original

For the cellular repair assay, cells were treated with KBrO_3_ or with Ro 19–8022 plus light and the removal of lesions during repair incubation was followed with time and is represented by t _1/2_ (Fig. 4 y-axis). For the in vitro repair assay, test cell extracts were incubated with substrate nucleoids from TK-6 cells that had been treated with either KBrO_3_ or with Ro 19–8022 plus light; incision (Fig. 4 x-axis) is expressed as increase in % tDNA after 1 h incubation. A high value of t_1/2_ (cellular repair assay) means slow repair, while a high in vitro incision rate means fast repair. No significant correlation was found between incision rate (in vitro repair assay) and t _1/2_ (cellular repair assay).

**Fig. 5:**
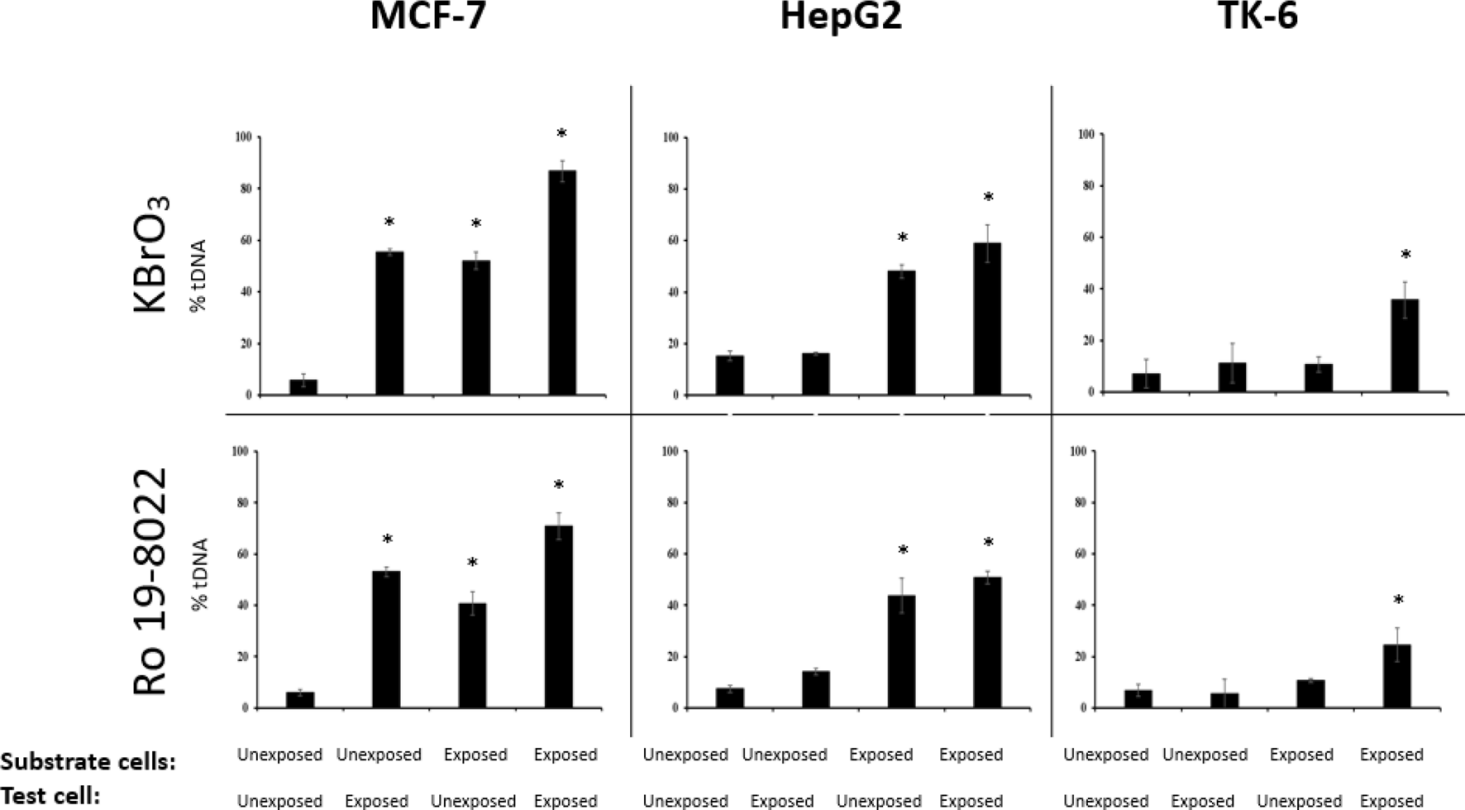
Incision in substrate nucleoids by extracts prepared from unexposed test cells or test cells exposed to KBrO_3_ (10 mM, 1 h) or to Ro 19–8022 (1 µM) plus light. The exposure status is indicated for each cell type in the lower lines of the figure, for both the substrate cells and the three different cell types used as source for repair extracts (from left to right, MCF-7, HepG2, and TK-6). Data are shown as mean values of three independent experiments (± SD indicated by error bars). Significantly different incision (by extracts from unexposed vs. exposed test cells) in substrate nucleoids (unexposed or exposed) is indicated by * (*P* ≤ 0.05). (Statistical significance was determined by comparing the values of % tDNA for each condition to the background level of the unexposed substrate (unexposed substrate treated with test cell extracts from unexposed cells.))

One important difference between the two assays is that cells are challenged with a genotoxicant in the cellular repair assay, whereas in the in vitro repair assay, the test cells (from which the repair extracts are made) are normally not additionally exposed. Therefore, the discrepancy between the two approaches might be explained by an effect of exposure to a DNA-damaging compound on at least the initial steps of BER. To test this hypothesis, we exposed batches of three cell types (MCF-7, HepG2, and TK-6) to KBrO_3_ or to Ro 19–8022 plus light, before preparing test cell extracts to be used in the in vitro repair assay. Indeed, the incision activity on TK-6 substrate nucleoids of the extracts from the pre-exposed test cells increased significantly, compared with the incision activity of extracts from unexposed test cells (*P* < 0.05) (Fig. 5). This was evident for pretreatments with both KBrO_3_ and with Ro 19–8022 plus light. There was no correlation between incision rates of extracts from unexposed test cells, and incision rates in extracts prepared from test cells exposed to KBrO_3_ or to Ro 19–8022 plus light (*R*^2^ = 0.11, *P* = 0.942 and *R*^2^ = 0.069, *P* = 0.569; respectively).

## Discussion

Various comet assay-based methods have been developed over the years to assess DNA repair activity. These methods have received attention because of their simplicity, low costs, and speed. The two approaches mostly used so far, and examined in the present study, comprise the cellular repair assay (a.k.a. challenge assay), and the in vitro repair assay that assesses the ability of test cell extracts to incise damaged DNA in substrate nucleoids. During the process of repair of DNA damage via BER, transient strand breaks and alkali labile sites are formed that can be detected by the comet assay (Collins and Azqueta 2014). In the in vitro repair process, incision (measured with the comet assay) is the endpoint, but in the cellular repair assay, the complete repair process, up to re-synthesis and ligation, is measured. The two assays, therefore, measure different endpoints, and there will not necessarily be a correlation when comparing these approaches in different cell types.

Although both assays have been considered to assess DNA repair activity, they were never directly compared to assess whether they give similar ranking of DNA repair activities for diverse cell types. Here we show that these two methods indeed do not correlate, and we suggest that this is due to the fact that they reflect different aspects of the complex multistep process of BER. We expected that the in vitro repair assay will only reflect the initial phases of BER (steps 1 and 2 as described in the Introduction), whereas the cellular repair assay reflects all four phases. However, DNA damage recognition and incision is considered to be the rate-limiting step in BER, and so it was expected that the results of both assays might still correlate. A more important difference between the two approaches is that cells need to be exposed to a DNA-damaging compound in the cellular repair assay, which can theoretically induce DNA repair activity, whereas cells whose extract is to be tested in the in vitro assay are not normally exposed to a genotoxin. In fact, exposure can change BER activity, as was shown before for various types of DNA lesions that are repaired by BER (Kassam and Rainbow 2008; Vidal et al. 2020; Vidal et al. 2018). We confirm here that incision of damaged DNA by cellular extracts is increased after exposure to the DNA-damaging compounds KBrO_3_ or Ro19-8022 plus light, both inducing DNA base oxidations. The underlying mechanism of this increased DNA repair activity after exposure to DNA-damaging compounds is not yet fully understood. In the case of exposure of test cells to Ro19-8022 plus light, the incision activity on damaged DNA in the in vitro repair assay was already increased after ~ 5 min of exposure in HepG2, TK-6, and MCF-7 cells. We previously showed that the induction of DNA repair after exposure to a DNA-damaging compound may be altered by the PI3K inhibitor 3-methyladenine (Schaaf et al. 2015), suggesting that the PI3K pathway may act as DNA damage sensor. These results indicate that the induction of BER after exposure to DNA-damaging compounds is relatively fast (within minutes) and is likely to involve activation of existing DNA repair proteins via DNA damage signaling, rather than the production of new DNA repair proteins via transcription and translation. We also showed recently that the induction of DNA repair may be different between cell lines and may depend on the amount of DNA damage that is present (Zheng et al. 2023). The repair activity assessed by the in vitro repair assay positively related to the background damage in the same cells. This suggests that the amount of ‘background’ DNA damage may also be a determinant for repair activity even without additional induction of damage by an external trigger.

Induction of DNA repair is thought to be the underlying mechanism of an adaptive response, and some studies have indicated that there may be cross-resistance; thus, exposure to one compound may improve the protection against another DNA-damaging compound. With the present in vitro repair assay, an increased incision rate of extracts from test cells whose DNA contained high levels of DNA oxidation damage was observed, indeed suggesting induction of DNA repair. Interestingly, also the incision of ‘undamaged’ control DNA was increased in extracts from MCF-7 cells but not in extracts from TK-6 or HepG-2 cells. This may reflect cross-resistance in which the DNA repair processes that are increased in MCF-7 cells may additionally recognize other (endogenous) lesions that are always present in DNA. However, this needs confirmation in additional studies, because the increase in ‘background’ incisions may also be caused by other processes leading to breaks or alkali labile sites. For instance, DNA breaks can be introduced by topoisomerases after oxidative stress (Li et al. 1999) or exposure may lead to the activation of endonucleases and caspases in the test cell extracts.

In conclusion, DNA repair is an important biomarker in clinical settings for predicting treatment outcomes, in human biomonitoring studies to understand inter-individual variation in levels of DNA damage and subsequent cancer risk, but also in experimental/mechanistic studies. The comet assay is easy to perform in such settings and actually both approaches used here have been applied previously although they have not been compared until now. In a recent analysis by the hComet consortium, data from DNA repair studies were pooled to investigate host factors that determine individual DNA repair ability (Azqueta et al. 2019). All of the studies in the pooled analysis used the in vitro repair assay, which is fortunate because we show here that comparing data from different comet assay-based approaches may actually not be valid. As demonstrated in the present study, result from the two assays does not correlate, probably because they measure different endpoints reflecting two different parts of the cellular DNA repair response. The in vitro repair assay seems to reflect the ongoing repair activity in the cells at the time the cells were collected, whereas the cellular challenge assay reflects the inducibility of DNA repair. Therefore, the assays should be seen as complementary, reflecting different phases in the complexity of the DNA repair response to protect genomic stability.

## Data Availability

All data generated or analyzed during this study are included in this published article.
